# Stepwise Achievement of Circularly Polarized Luminescence on Atomically Precise Silver Clusters

**DOI:** 10.1002/advs.202000738

**Published:** 2020-06-11

**Authors:** Si Li, Zhi‐Ping Yan, Xin‐Lei Li, Yu‐Jin Kong, Hai‐Yang Li, Guang‐Gang Gao, You‐Xuan Zheng, Shuang‐Quan Zang

**Affiliations:** ^1^ Green Catalysis Center and College of Chemistry Zhengzhou University Zhengzhou 450001 China; ^2^ State Key Laboratory of Coordination Chemistry School of Chemistry and Chemical Engineering Nanjing University Nanjing 210023 China; ^3^ School of Materials Science and Engineering University of Jinan Jinan 250022 China

**Keywords:** chirality, circularly polarized luminescence, postmodification, precise silver clusters, silver (I) clusters

## Abstract

The weakly coordinated anionic nitrate ligands in a centrosymmetric Ag_20_ cluster are replaced in a stepwise manner by chiral amino acids and two achiral luminescent sulfonic‐group‐containing ligands while nearly maintaining the original silver(I) cage structure. This surface engineering enables the atomically precise Ag_20_ clusters to exhibit the high‐efficiency synergetic effects of chirality and fluorescence, producing rare circularly polarized luminescence among the metal clusters with a large dissymmetry factor of (|g_lum_|) ≈ 5 × 10^−3^. This rational approach using joint functional ligands further opens a new avenue to diverse multifunctional metal clusters for promising applications.

## Graphical abstract

A step‐by‐step surface modification approach is proposed to synthesize multifunctional silver(I) nanoclusters. By combining both chiral and luminescent organic ligands on the Ag_20_ surface, the circularly polarized luminescence reactivity is rationally designed for silver(I) cluster compounds. This rational approach using joint functional ligands further opens a new avenue to diverse multifunctional metal clusters for promising applications.

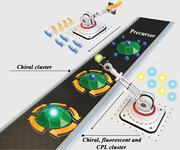

Atomically precise silver(I) nanoclusters have attracted much attention due to their diverse structures and potential applications in luminescence, sensors, and bioactive materials.^[^ ^1^, ^2^, ^3^, ^4^ ^]^ The syntheses of these structure‐defined silver(I) nanoclusters are usually achieved by a molecular self‐assembly of organic ligands with different silver(I) salts, where the structures and functions can be adjusted by the selection of the template, auxiliary ligand, or reaction conditions.^[^ ^5^, ^6^, ^7^ ^]^ To date, a number of Ag(I)‐nanocluster compounds have been determined, and their structures can be modulated by Ag—S, Ag—C, or Ag—Ag bonds.^[^ ^5^, ^6^, ^7^ ^]^ However, most atomically precise Ag(I) clusters are usually formed by the self‐assembly of specific ligands such as alkynyl and thiolate organic ligands, which limits further postmodification and the available functionality. Thus, the rational design and targeting synthesis of functional silver(I) clusters still require the development of a new synthetic method.^[^ ^8^ ^]^ The current synthetic approach used for Ag(I) clusters usually employs the reaction of silver(I)‐alkynyl or silver(I)‐thiolate oligomers dissolved in condensing silver(I) salt solution. With such a technique, the reaction only depends on the self‐assembly process, and the final cluster structure cannot be predicted and designed. Sporadic silver(I) clusters can also be synthesized by the solventothermal technique, but the reaction occurs in a “black box” and the product is uncontrollable.^[^ ^6^ ^]^ Thus, a self‐assembly‐based synthetic approach should be developed for the rational design of functional silver(I) cluster compounds.

Recently, we unveiled that the Ag_20_ nanocluster is a thermodynamically stable species in the self‐assembly process, in which weak surface coordination ligands, such as NO_3_^−^ and dimethylacetamide, can be substituted by strong coordination ligands and thus facilitate a new one‐step postmodification technique for the molecular design of new silver(I) cluster compounds.^[^ ^8b^ ^]^ With this one‐step substitution, parts of weak surface ligands can be replaced by different functional molecules, where the chirality or luminescence is incorporated into the Ag_20_ cluster matrix. However, such a one‐step substitution can only realize a single function. From the viewpoint of functional materials, an application often requires the combination of multiple functions to achieve synergistic effects. Therefore, for the synthetic technique, the one‐step surface postmodification should be expanded for silver(I) clusters. If the surface modification can be achieved in a step‐by‐step manner, the synthesis of functional silver clusters will enter into a new modular assembly stage by virtue of the central silver(I) core and its surface functional ligands as secondary building blocks. Considering that Ag_20_ nanoclusters still possess weak surface ligands after the one‐step modification, we believe that a step‐by‐step modification is feasible for the proposed surface postmodification.

Circularly polarized luminescence (CPL) originates from the chiral emissive states of luminescent materials, which inherently requires a efficient synergy between luminescence and chirality. CPL are attracting interests in stereo‐imaging, security encoding, optical data storage, and 3D displaying.^[^ ^9^ ^]^ However, it is still a great challenge to achieve either bright room‐temperature luminescence or chirality on an atomically precise silver cluster by far, and the CPL is all the more difficult since the low photoluminescence quantum yield and the small dissymmetry factors hinder the detection of CPL. One feasible way to develop new CPL‐active materials is to design a “host‐guest” hybrid system in which luminescent guests are entrapped in the chiral host. The junction of the host and guest molecules can lead to CPL activity for these materials.^[^ ^10^ ^]^ This effective approach inspired us to obtain unexplored silver(I)‐cluster‐based CPL materials by further combining chiral and luminescent ligands. Considering that the surface of a one‐step‐modified Ag_20_ cluster still contains weak ligands, we anticipate that the chiral and luminescent ligands can be both anchored on the Ag_20_ cluster by a two‐step process. Then, the combination of chirality and luminescence may induce CPL activity that the combined function has never been explored for silver(I) nanoclusters.

In this work, a 20‐core thiolate Ag(I) cluster matrix is first postmodified by chiral amino acids and forms the chiral Ag_24_ cluster expressed as (CO_3_)@Ag_24_(S*^t^*Bu)_10_(L/D‐Proline)_8_(NO_3_)_2_·(NO_3_)_2_ (L/D‐Ag_24_), as determined in our recent work.^[^ ^8b^ ^]^ Four NO_3_^−^ residues in L/D‐Ag_24_ facilitate the further substitution by other ligands. By the substitution of an elaborate choice of luminescent ligands of 4,4′‐bis(2‐sulfonatostyryl)biphenyl disodium salt (Na_2_CF351) and *N*,*N*′‐di(ethanesulfonic acid)‐3,4,9,10‐perylene tetracarboxylic diimide (H_2_TauPDI) (Figure S1, Supporting Information), new chiral‐luminescent compounds of (CO_3_)@Ag_24_(S*^t^*Bu)_10_(L/D‐Proline)_8_(CF351)_2_ (**L‐CF351** or **D‐CF351**) and (CO_3_)@Ag_24_(S*^t^*Bu)_10_(L/D‐Proline)_8_(TauPDI)_2_ (**L‐TauPDI** or **D‐TauPDI**) are demonstrated, which present interesting CPL behavior and confirm the feasibility of the step‐by‐step postmodification approach (**Scheme** **1**).

**Scheme 1:**
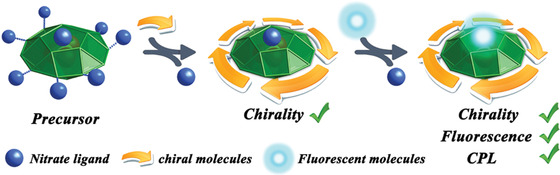
The proposed synthetic pathways for multi‐functional silver(I) clusters.

In the syntheses of compounds **L/D‐CF351** and **L/D‐TauPDI**, two kinds of routes are effective in obtaining the targeting products. The L/D‐Ag_24_ complex was first synthesized by a one‐step substitution under the optimized conditions. Then, the solution of Na_2_CF351 or H_2_TauPDI was spread out on the surface of the L/D‐Ag_24_ solution. By liquid‐liquid diffusion, crystals of **L/D‐CF351** or **L/D‐TauPDI** successfully formed on the two‐phase interface (Method A in the Supporting Information, Figures S2 and S3, Supporting Information). Such a synthetic process undoubtedly confirms the feasibility of the two‐step postmodification approach. However, the calculated yields based on Ag_24_ starting materials are only approximately 10%. Thus, we also explored the synthesis of **L/D‐CF351** or **L/D‐TauPDI** by an in situ two‐step approach, in which L/D‐Ag_24_ is not isolated from the solution and the solution directly reacts with Na_2_CF351 or H_2_TauPDI in the second reaction process (Method B in the Supporting Information). Fortunately, by the in situ two‐step postmodification process, we obtained products with a higher yield (≈65% for **L/D‐CF351** and ≈22% for **L/D‐TauPDI**). The above synthetic processes both unveil that the step‐by‐step postmodification is effective in producing multifunctional silver(I) cluster compounds.

In addition, in our previous attempt, fluorescent ligands with carboxylate group were explored to accomplish the second modification. Unfortunately, both aromatic and alkane carboxylate ligands exhibit a strong coordination ability that can competitively replace the amino acids and NO_3_^−^ ligands to form the simplex modification products (Figure S4, Supporting Information). This result relates to the fact that the pKa values of L/D‐proline and NO_3_^−^ ligands are 1.99 and −2, respectively, so the pKa value of the luminescent ligands should be in the range from 1.99 to −2. However, the pKa values of aromatic carboxylic acids are far greater than 1.99 (the pKa of benzoic acid is 4.2), which results in the complete replacement of L/D‐proline and NO_3_^−^ ligands. Fortunately, we found that the pKa values of phenyl sulfonic acid (0.7) and taurine (1.5) are in the above range, so the elaborate choice of H_2_TauPDI or Na_2_CF351 with sulfonic groups facilitates the replacement of NO_3_^−^ weak ligands without affecting the coordinated amino acids anchored in the first step.

The chiral nanocluster of L/D‐Ag_24_ has been determined by single‐crystal structure analysis in our group (Figure S5, Supporting Information). Ten S*^t^*Bu ligands stabilize the 20‐core silver(I) cluster by diverse Ag—S bonds, in which the Ag^+^ ions can be divided into one large Ag_10_ circle and two small Ag_5_ circles. Eight chiral proline ligands are linked to the silver(I) cluster by Ag—O bonds (2.241–2.436 Å, Figure S6, Supporting Information) between the carboxylate oxygen and Ag^+^ ion from the Ag_10_ circle. The neighboring proline ligands are further linked by a Ag^+^ ion to form a chiral dimer, and the whole molecule looks like a propeller. Compared with NO_3_^−^ ligands (Ag—O bond lengths are 2.308–2.696 Å, Figure S6, Supporting Information), the stronger coordination ability of the chiral dimers leads to an asymmetric tensile deformation of the Ag_10_ circle from a round shape to an oval shape (the central carbon atom from CO_3_^2−^ is the benchmark, Figure S7, Supporting Information), and the length and width are 10.669 and 9.980 Å, respectively. At the same time, the distance and angle between the Ag_5_ circles become 3.489 Å and 2.138^o^, respectively, accompanied by an asymmetric contraction of the Ag_5_ circles. All of these changes induce the generation of a pair of chiral Ag cores (Figures S8 and S9, Supporting Information). In addition, four NO_3_^−^ ions are used to balance the charge that could be substituted by other ligands. In the compounds of **L/D‐CF351** and **L/D‐TauPDI**, the structural analysis indicates that the L/D‐Ag_24_ cluster maintains its basic feature (chiral dimers and a chiral core, detailed bond lengths are shown in Table S3‐S5), except that the four NO_3_^−^ ions have been substituted by sulfonic groups (**Figure** **1** and Figure S10, Supporting Information). The oxygen atoms of the sulfonic group link the Ag^+^ ions from the Ag_5_ circles, in which the Ag—O bonds are in the range of 2.524–2.749 Å for **L/D‐CF351** and 2.525–2.803 Å for **L/D‐TauPDI** (Figures S11 and S12, Supporting Information). These Ag—O bonds solidify the linkage between the ligands and the Ag_20_ core. The S⋅⋅⋅S distances between the two sulfur atoms on top of the Ag_5_ circles are ≈8.100 Å for **L/D‐CF351** and ≈8.408 Å for **L/D‐TauPDI**, indicating the different interactions between the luminescent ligands and the central Ag_20_ core. This phenomenon may be ascribed to the intermolecular interactions. The F‐type stacking interaction (vertical distance of ≈3.349 Å and face center distances of 3.694–3.874 Å) in **L/D‐TauPDI** is stronger than the few C—H⋅⋅⋅*π* interactions in **L/D‐CF351** (Figures S13 and S14, Supporting Information). In addition, all the structures belong to the chiral *P1* space group, and Flack parameters less than 0.06 suggest a homochiral molecular packing of these crystals. To the best of our knowledge, these compounds are the first examples of both chiral and luminescent ligands being simultaneously modified on a silver(I) cluster surface.

**Figure 1:**
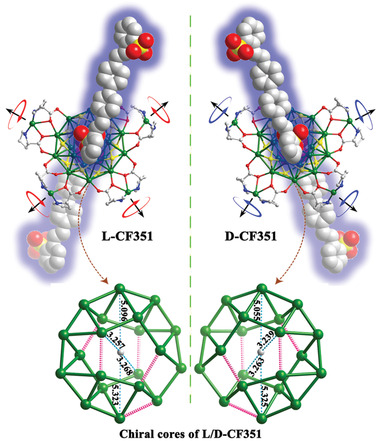
Crystal structures of enantiomeric **L/D‐CF351** (top); all H atoms, the *tert*‐butyl groups and free nitrates are omitted for clarity. The chiral Ag cores of **L/D‐CF351** (bottom); the unit of the numbers is Å, and the pink dotted lines indicate that the Ag···Ag distances are longer than 3.44 Å. Color code: Ag, green; S, yellow; O, red; N, blue; C, gray.

The UV–vis absorption spectra indicate that **L/D‐CF351** and **L/D‐TauPDI** absorb both UV and visible light with wavelengths from 240 to 800 nm in the solid state (Figure S15, Supporting Information). Thus, circular dichroism (CD) spectra were detected in the same range. L/D‐proline and L/D‐Ag_24_ only present absorbance in the UV band (**Figure** **2**a,b). In contrast, both **L/D‐CF351** and **L/D‐TauPDI** display an intense cotton effect and an excellent mirror image relationship in the 250–500 nm and 300–700 nm ranges, respectively, which correspond to their absorption spectra. Therefore, the CD signals of **L/D‐CF351** and **L/D‐TauPDI** in the visible region can be ascribed to the chirality transfer from the chiral silver(I) cluster to the originally achiral luminescent ligands. From an analysis of the CF351^2−^ molecular configuration in **L/D‐CF351**, it is found that the CF351^2−^ molecules are induced to chiral configurations (Figure S16, Supporting Information). Additionally, the C—H⋅⋅⋅*π* interactions between the *tert*‐butyl groups or proline molecules and CF351^2−^ ligands are negligible (longer than 4 Å, Figure S17, Supporting Information), so the CD responses of the CF351^2−^ molecules are mainly due to the core‐to‐ligand chirality transfer. Similar to the CF351^2−^ molecules, the TauPDI^2−^ molecules in **L/D‐TauPDI** show chiral Gauche^+^ and Gauche^−^ configurations. And there are many C—H⋅⋅⋅*π* interactions between the *tert*‐butyl groups or proline molecules and the TauPDI^2−^ ligands (Figures S18 and S19, Supporting Information), so the CD responses are related to the core‐to‐ligand and ligand‐to‐ligand chirality transfer. We have also found another commonality in **L/D‐CF351** and **L/D‐TauPDI**, where the molecular groups far away from the chiral Ag_24_ cluster are not induced to chiral configurations, the phenyl sulfonic and ethyl sulfonic groups exhibit achiral configurations, indicating that the chirality transfer may also induced by chiral spaces on both the top and bottom of the Ag_24_ propellers.

**Figure 2:**
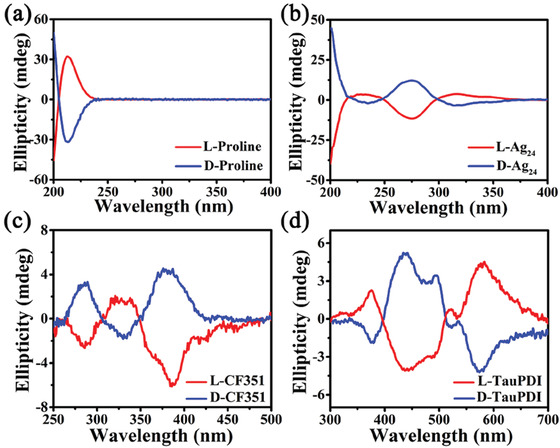
CD spectra for a) L/D‐proline in water, b) L/D‐Ag_24_ in ethanol, and c) **L/D‐CF351** and d) **L/D‐TauPDI** in the solid state at room temperature.

As shown in **Figure** **3**a–d, **L‐CF351** exhibits blue emission around 458 nm in the solid state, and the water/methanol dispersion solution of **L‐TauPDI** exhibits yellow emission around 535 and 571 nm (H_2_TauPDI is an aggregation‐caused quenching (ACQ) molecule). The nanosecond lifetimes (*τ* **_L‐CF351_** = 0.8 ns, *τ* **_L‐TauPDI_** = 4.2 ns) of **L‐CF351** and **L‐TauPDI** indicate that the excited states responsible for luminescence are singlet in origin. Furthermore, the similar photophysical parameters of **L‐CF351**, **L‐TauPDI,** and the pure luminescent ligands (*λ*_Na2CF351_ = 477 nm, *τ*_Na2CF351_ = 3.4 ns; *λ*_H2TauPDI_ = 545 nm, *τ*_H2TauPDI_ = 5.8 ns) indicate that the emissions of the clusters are mainly ascribed to the luminescent ligands. To determine whether the dispersion of **L/D‐TauPDI** in the water/methanol solution damages the chirality transfer pathway, the CD property of the dispersion solution was also explored. As shown in Figure S20, Supporting Information, the dispersion solutions of **L/D‐TauPDI** display an excellent mirror image relationship in the 260–600 nm range, which corresponds to the absorption spectra, indicating that the chirality transfer process still exists. The change in the CD spectra may be caused by the weakness of the *π*‐*π* stacking interactions between the TauPDI^2−^ ligands in the water/methanol solution. Considering that both the chiral and luminescent ligands coordinate to the Ag_20_ cluster and the chirality transfer process, the CPL properties were also explored. As shown in Figure 3e,f, the chiral silver(I) clusters show a strong CPL response in the same wavelength region as their emission bands with a different handedness. The calculated values of the dissymmetry factor (|*g*_lum_|) of the CPL signals are ≈5 × 10^−3^ and ≈4 × 10^−3^, respectively (Figures S21 and S22, Supporting Information). Thus, the synergy between endowed chirality and inherent emitting ability, generating the scarcely bright CPL with high dissymmetry factors. This adjustable CPL activity is first observed in the cluster chemistry, which encourages us to achieve full‐color covering CPL systems through the step‐by‐step surface modification. Furthermore, our proposed method is suitable for different luminescent molecules (Aggregation‐induced emission or ACQ molecules), which could be widely used to fabricate cluster‐based CPL‐active materials.

**Figure 3:**
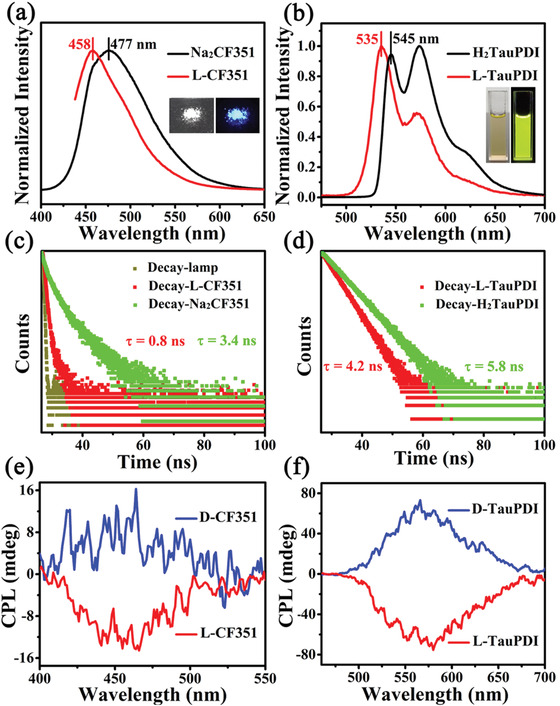
a) Fluorescence spectra of Na_2_CF351 (black curve) and **L‐CF351** (red curve) in the solid state, excited at 371 and 420 nm, respectively. b) Fluorescence spectra of the water/methanol dispersion solution of H_2_TauPDI (black curve) and **L‐TauPDI** (red curve), excited at 369 nm; the insets show the corresponding photographs of **L‐CF351** (solid state) and **L‐TauPDI** (dispersion solution) under light field and under 365 nm UV irradiation, respectively. c) Luminescence decay traces of **L‐CF351** (red block, at 458 nm) and Na_2_CF351 (green block, at 477 nm) in the solid state after excitation at 405 nm. d) Luminescence decay traces of **L‐TauPDI** (red block, at 535 nm) and H_2_TauPDI (green block, at 545 nm) in the water/methanol solution after excitation at 455 nm. e,f) CPL spectra of **L/D‐CF351** enantiomers in the solid state and the water/methanol dispersion solution of **L/D‐TauPDI**.

In conclusion, a new step‐by‐step surface modification approach has been proposed to synthesize multifunctional silver(I) nanoclusters. By combining both chiral and luminescent organic ligands on the Ag_20_ surface, the CPL reactivity has been rationally designed for silver(I) cluster compounds. This work not only extends a new approach to synthesizing multifunctional silver(I) nanoclusters but also sheds light on the new atom‐level design of CPL materials based on silver(I) host‐guest molecules.

[CCDC 1966243, 1966244, 1966245, and 1966246 contain the supplementary crystallographic data for this paper. These data can be obtained free of charge from The Cambridge Crystallographic Data Centre via www.ccdc.cam.ac.uk/data_request/cif.]

## Conflict of Interest

The authors declare no conflict of interest.

## Supporting information

Supporting Information
