# Supplementary material for: Stepwise Achievement of Circularly Polarized Luminescence on Atomically Precise Silver Clusters
Source: Adv Sci (Weinh). 2020 Jun 11;7(15):2000738. doi: 10.1002/advs.202000738 (PMC7404152; doi:10.1002/advs.202000738)
Supplement: Supplementary file 1 — Supporting Information [file ADVS-7-2000738-s001.pdf]

© 2020 Wiley-VCH GmbH

ADVANCED SCIENCE
Open Access

# Supporting Information

for *Adv. Sci.*, DOI: 10.1002/advs.202000738

# Stepwise Achievement of Circularly Polarized Luminescence on Atomically precise Silver Clusters

*Si Li,[#] Zhi-Ping Yan,[#] Xin-Lei Li, Yu-Jin Kong, Hai-Yang Li, Guang-Gang Gao,* You-Xuan Zheng, and Shuang-Quan Zang**

WILEY-VCH

Copyright WILEY-VCH Verlag GmbH & Co. KGaA, 69469 Weinheim, Germany, 2020.

# Supporting Information

**Stepwise Achievement of Circularly Polarized Luminescence on Atomically precise Silver Clusters**

*Si Li,[#] Zhi-Ping Yan,[#] Xin-Lei Li, Yu-Jin Kong, Hai-Yang Li, Guang-Gang Gao,* You-Xuan Zheng, and Shuang-Quan Zang**

## Experimental Section

**Materials and Reagents**. All chemicals and solvents obtained from suppliers were used without further purification. All solvents were analytical grade reagent.

**Instrumentation**. The X-ray powder diffraction (PXRD) patterns of all compounds were collected at room temperature in air on an X'Pert PRO diffractometer (Cu-K$\alpha$). The UV-vis absorption spectra were recorded using a Hitachi UH4150 UV-visible spectrophotometer in the wavelength range of 200-800 nm. The emission and excitation spectra were measured at room temperature with a HORIBA FluoroLog-3 fluorescence spectrometer. The luminescence lifetime was measured on a HORIBA FluoroLog-3 fluorescence spectrometer equipped with a 405 or 455 nm laser operating in time-correlated single photon counting mode (TCSPC) with a resolution time of 200 ps. The circular dichroism (CD) spectra were recorded by a Chirascan V100 spectropolarimeter in a water/methanol solution or with pressed KBr pellets at a scan speed of 100 nm·min$^{-1}$ with a bandwidth of 1 nm. Circularly polarized luminescence (CPL) measurements were performed with a JASCO CPL-300 spectrometer. CPL spectra of samples were measured at room temperature by using 1 cm × 1 mm (optical path 1mm) cuvettes or with pressed KBr pellets over a range of 400-700 nm. The maximum emission peaks were modulated around 0.5 V, and the excitation wavelengths were 371 nm and 420 nm, respectively. The value of $g_{\text{lum}}$ was defied as $g_{\text{lum}}$ = 2 × [ellipticity/(32980/ln10)]/total florescence intensity at the CPL extremum. The samples were fixed on instrument directly and perpendicular to the light beam during the CD and CPL measurement. The background spectra from the quartz glass substrates were subtracted to obtain the final processed CD and CPL spectra.

**X-Ray Crystallography** Single-crystal X-ray diffraction measurements of the complexes **L/D-CF351** and **L/D-TauPDI** were performed with a Rigaku XtaLAB Pro diffractometer with Cu K$\alpha$ radiation ($\lambda$ = 1.5418 Å). The data collection and reduction were performed using the program CrysAlisPro.[S1] The data collection and reduction were performed using the program SADABS.[S2] All the structures were solved with direct methods (*SHELXS*)[S3] and refined by full-matrix least squares on $F^2$ using *OLEX2*,[S4] which utilizes the *SHELXL*-2015 module.[S5] All the atoms were refined anisotropically. The hydrogen atoms were placed at calculated positions refined using idealized geometries and assigned fixed isotropic displacement parameters. The structure refinement was handled with different strategies according to the electron density distribution and the complexity of the disorder. The imposed restraints and constraints (ISOR, DFIX, SIMU, SADI, DANG, etc.) in the least-squares refinement of each structure were commented in the corresponding crystallographic CIF files. A satisfactory disorder model for the solvent molecules was not found in **L/D-CF351** and **L/D-TauPDI**; therefore, the *OLEX2* Solvent Mask routine (*PLATON/SQUEEZE*)[S6] was used to mask out the disordered density.

The detailed information of the crystal data, data collection and refinement results for all compounds are summarized in Tables S1 and S2.

**Optimized conditions for Synthesis of L/D-$Ag_{24}$.**

A mixture of 0.05 mmol $[AgS^tBu]_n$ precursor with 0.05 mmol $AgNO_3$ in 2 mL acetonitrile was treated under ultrasonic conditions until a clear solution was obtained. A freshly prepared solution of L-proline or D-proline (12 mg, 0.1 mmol in 240 $\mu$L of water) was added to the above colorless solution under vigorous stirring at room temperature, followed by the addition of triethylamine (50 $\mu$L), after which the clear colorless solution became cloudy immediately. Then, extra silver nitrate (0.1 mmol) was added to the suspension solution, which turned into a clear colorless solution. The resultant solution (**A**) was allowed to evaporate slowly in

darkness at room temperature for two days to produce colorless block crystals. The yields were 72.5% (**L-Ag$_{24}$**) and 74.6% (**D-Ag$_{24}$**) based on Ag. The elemental analysis calculation (%) for $C_{89}H_{174}N_{16}S_{10}Ag_{24}O_{35}$ (4937.91) was C 21.65, H 3.55, N 4.54, and S 6.49; the measured result was C 21.55, H 3.46, N 4.41, and S 6.55.

**Synthesis of L/D-CF351 or L/D-TauPDI.**

**Method A: L/D-Ag$_{24}$** (10 mg) was dissolved in a methanol-water solution (2 mL, v:v = 3:1) and placed at the bottom of a test tube. Then, a methanol solution (2 mL) of $Na_2$CF351 (4 mg) was spread out on the surface of the above solution. The test tube was sealed in darkness at room temperature for two days to produce colorless strip crystals at the two-phase interface. The yields were 12.6% (**L-CF351**) and 14.3% (**D-CF351**) based on Ag. The elemental analysis calculation (%) for $C_{137}H_{196}Ag_{24}N_8O_{32}S_{14}$ (5504.73) was C 29.89, H 3.59, N 2.04, and S 8.15; the measured result was C 29.37, H 3.64, N 1.83, and S 7.99. The synthetic method of **L/D-TauPDI** was similar to that of **L/D-CF351**, except that the ligand was changed to $H_2$TauPDI (2 mg in 2 mL of methanol). The yields were 8.5% (**L-TauPDI**) and 7.9% (**D-TauPDI**) based on Ag. The elemental analysis calculation (%) for $C_{137}H_{188}Ag_{24}N_{12}O_{40}S_{14}$ (5680.76) was C 28.96, H 3.34, N 2.96, and S 7.90; the measured result was C 29.21, H 3.39, N 2.74, and S 7.89.

**Method B:** A freshly prepared **A** solution was combined with a methanol solution (1 mL) of $Na_2$CF351 (6 mg); then, the mixed solution was allowed to evaporate slowly in darkness at room temperature for two days to produce colorless strip crystals. The yields were 62.8% (**L-CF351**) and 67.3% (**D-CF351**) based on Ag. The synthetic method of **L/D-TauPDI** was similar to that of **L/D-CF351**, except that the ligand was changed to $H_2$TauPDI (2 mg in 1 mL of methanol). The resultant red dispersion solution was allowed to evaporate slowly in darkness at room temperature for two days to produce dark red block crystals. The yields were 21.6% (**L-TauPDI**) and 22.3% (**D-TauPDI**) based on Ag.

## Selected spectra and data referred in the paper

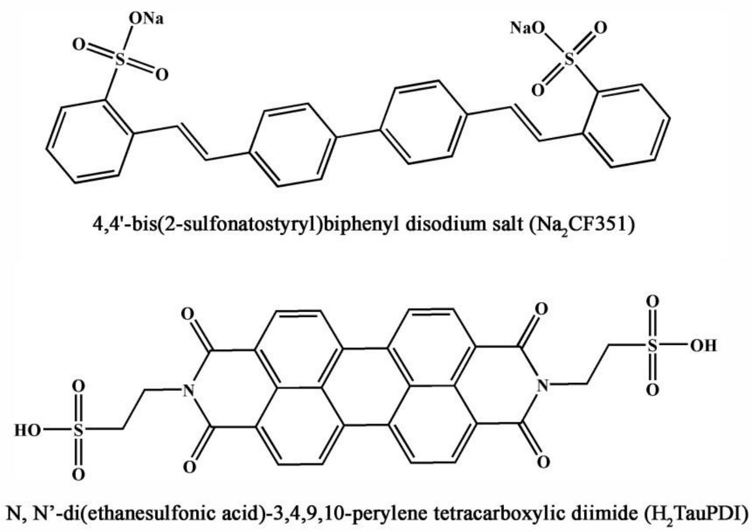


**Figure S1.** Molecular structures of **$Na_2$CF351** and **$H_2$TauPDI**.

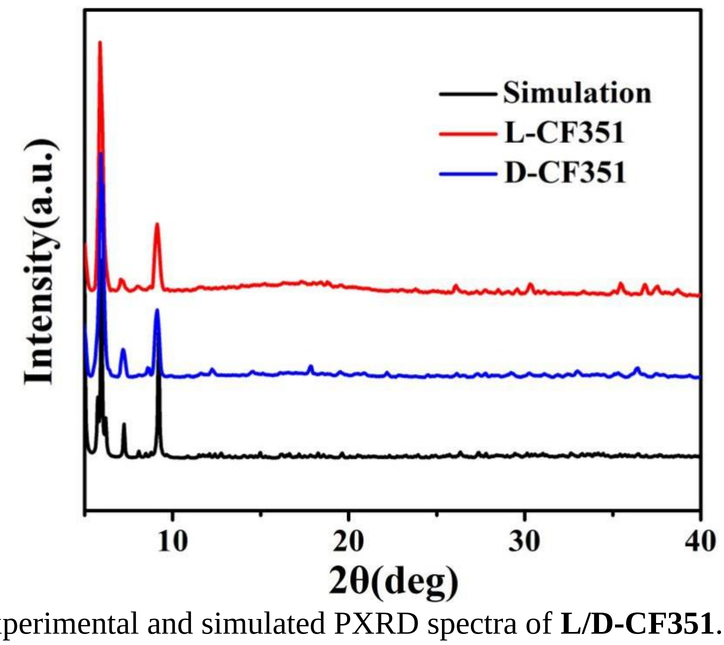


**Figure S2.** The experimental and simulated PXRD spectra of **L/D-CF351**.

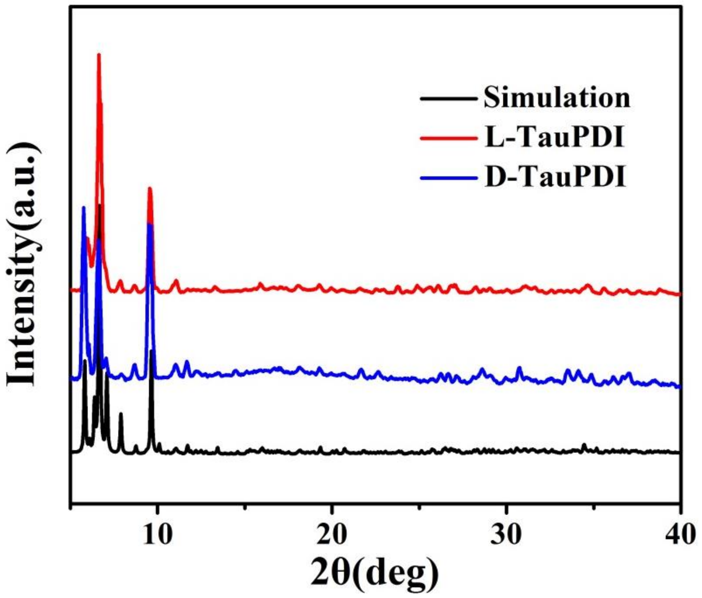


**Figure S3.** The experimental and simulated PXRD spectra of **L/D-TauPDI**.

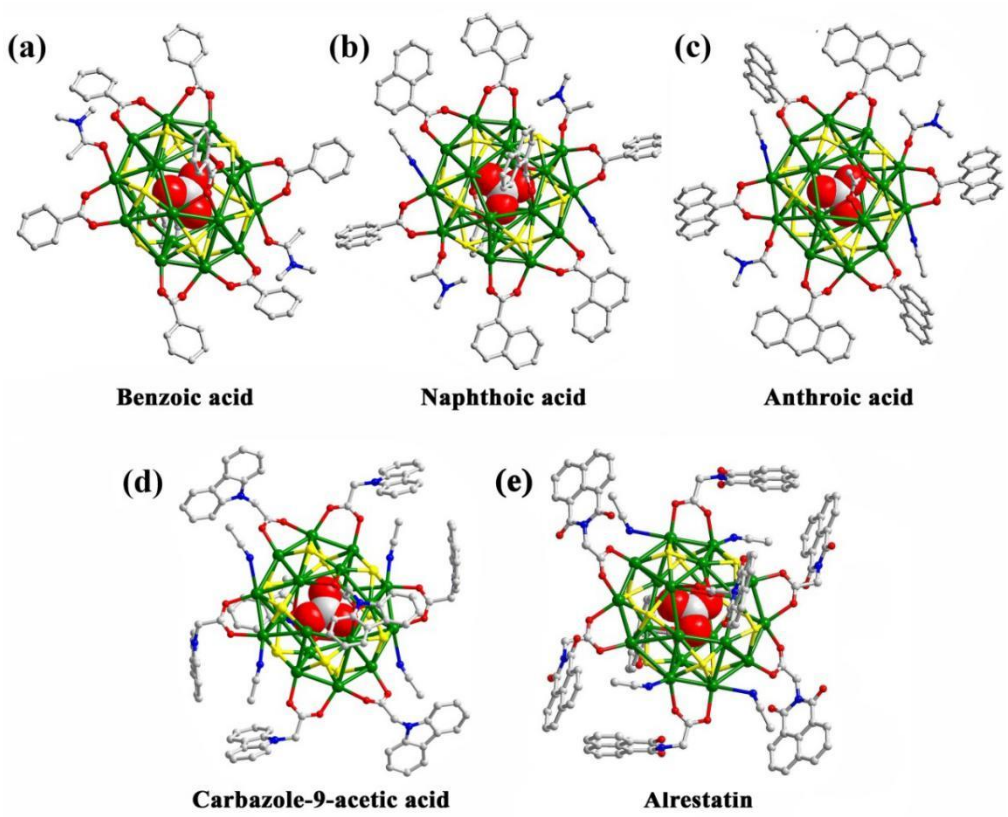

**Figure S4.** The simplex modification clusters produced by replacing the chiral amino acid and $NO_3^-$ ligands with benzoic acid (a), naphthoic acid (b), anthroic acid (c), carbazole-9-acetic acid (d) and alrestatin (e). All H atoms and the *tert*-butyl groups are omitted for clarity. Color code: Ag, green; S, yellow; O, red; N, blue; C, gray.

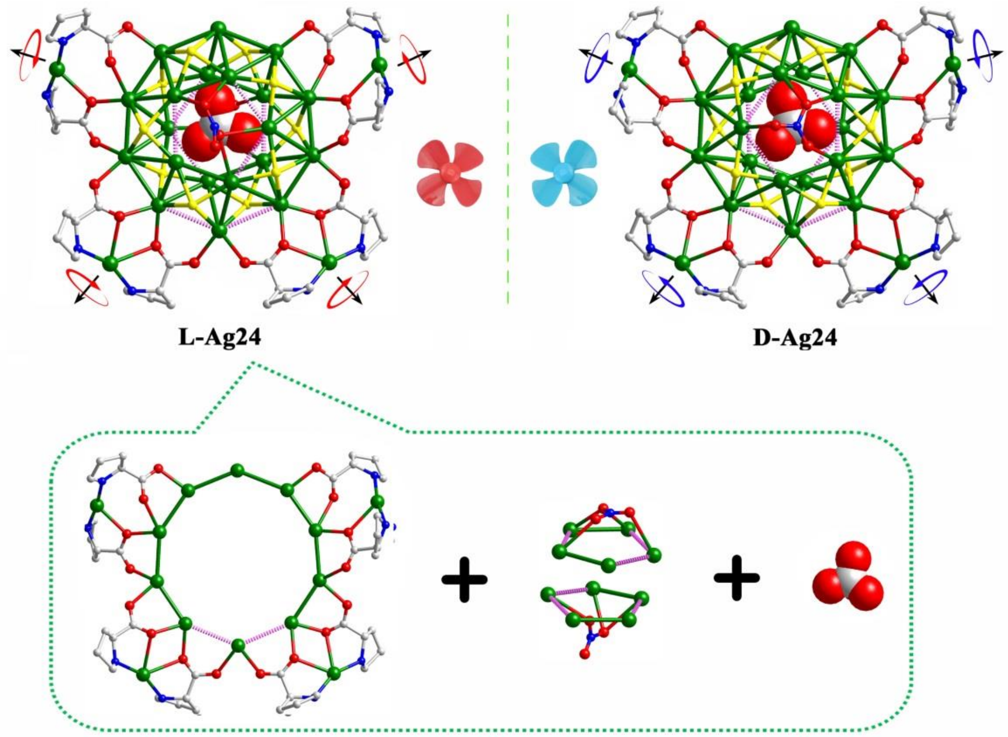


**Figure S5**. Ball-stick representation of **L/D-Ag24** and the anatomy of **L-Ag24**. All H atoms, the *tert*-butyl groups and free nitrates are omitted for clarity, and the pink dotted lines indicate that the Ag···Ag distances are longer than 3.44 Å. Color code: Ag, green; S, yellow; O, red; N, blue; C, gray.

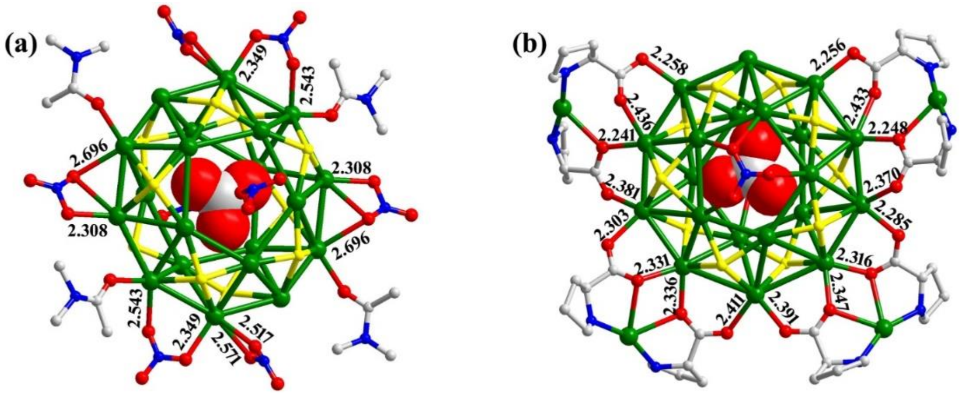

**Figure S6.** Detailed Ag–O bond lengths in $Ag_{20}$ precursor (a) and L-$Ag_{24}$ (b), the unit of the numbers is Å.

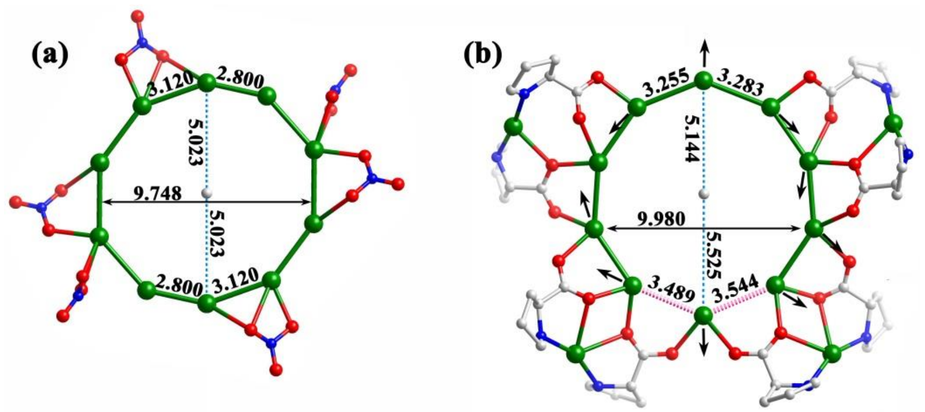


**Figure S7.** Ball-stick representation of the $Ag_{10}$ circles in the precursor (a) and L-$Ag_{24}$ (b); the modification of L/D-proline leads to an asymmetric tensile deformation of the $Ag_{10}$ circle from a round shape to an oval shape (the central carbon atom from $CO_3^{2-}$ is the benchmark). The unit of the numbers is Å, and the pink dotted lines indicate that the Ag···Ag distances are longer than 3.44 Å. Color code: Ag, green; O, red; N, blue; C, gray.

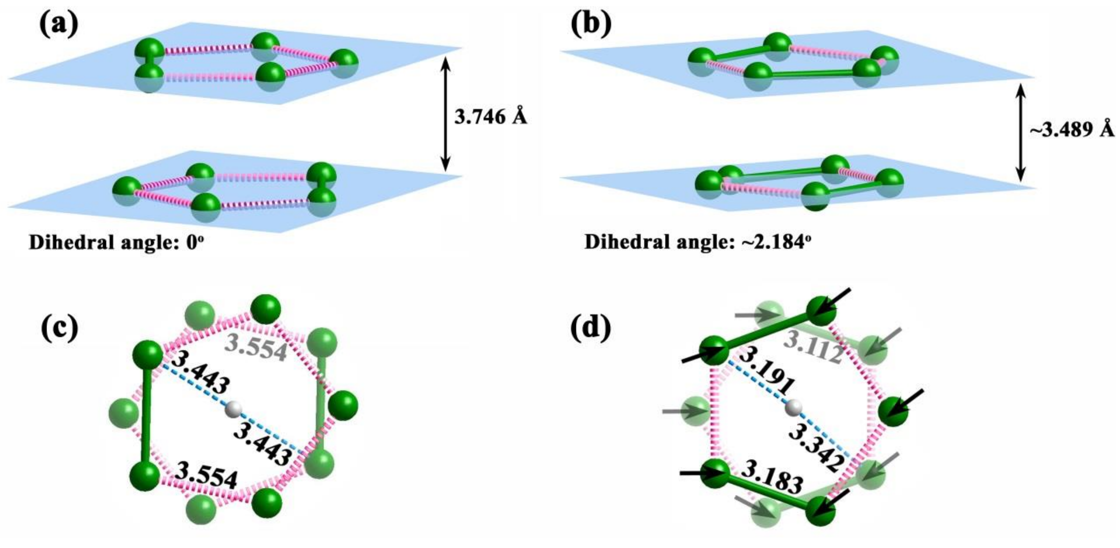

**Figure S8.** Distance and angle between the $Ag_5$ circles in the precursor (a) and L-$Ag_{24}$ (b), and a ball-stick representation of the $Ag_5$ circles in the precursor (c) and L-$Ag_{24}$ (d). The unit of the numbers is Å, and the pink dotted lines indicate that the Ag···Ag distances are longer than 3.44 Å.

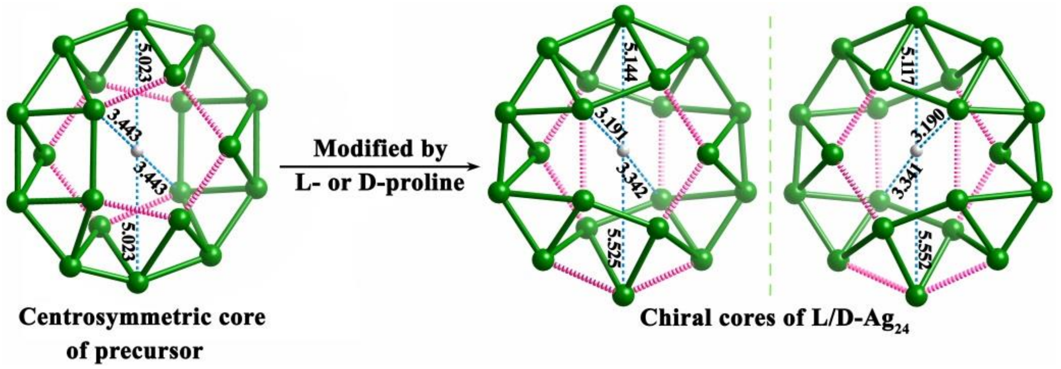


**Figure S9.** Ball-stick representation of the centrosymmetric Ag core of the precursor (left) and a pair of chiral Ag cores of L/D-$Ag_{24}$ (right). The unit of the numbers is Å, and the pink dotted lines indicate that the Ag···Ag distances are longer than 3.44 Å.

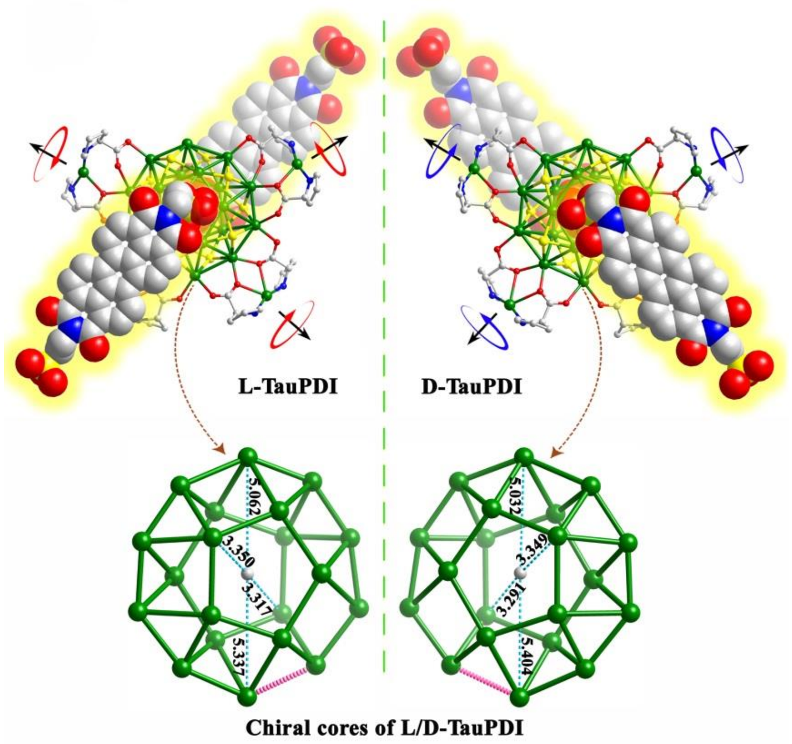


**Figure S10.** Crystal structures of enantiomeric **L/D-TauPDI** (top); all H atoms, the *tert*-butyl groups and free nitrates are omitted for clarity. The chiral Ag cores of **L/D-TauPDI** (bottom);

the unit of the numbers is Å, and the pink dotted lines indicate that the Ag···Ag distances are longer than 3.44 Å. Color code: Ag, green; S, yellow; O, red; N, blue; C, gray.

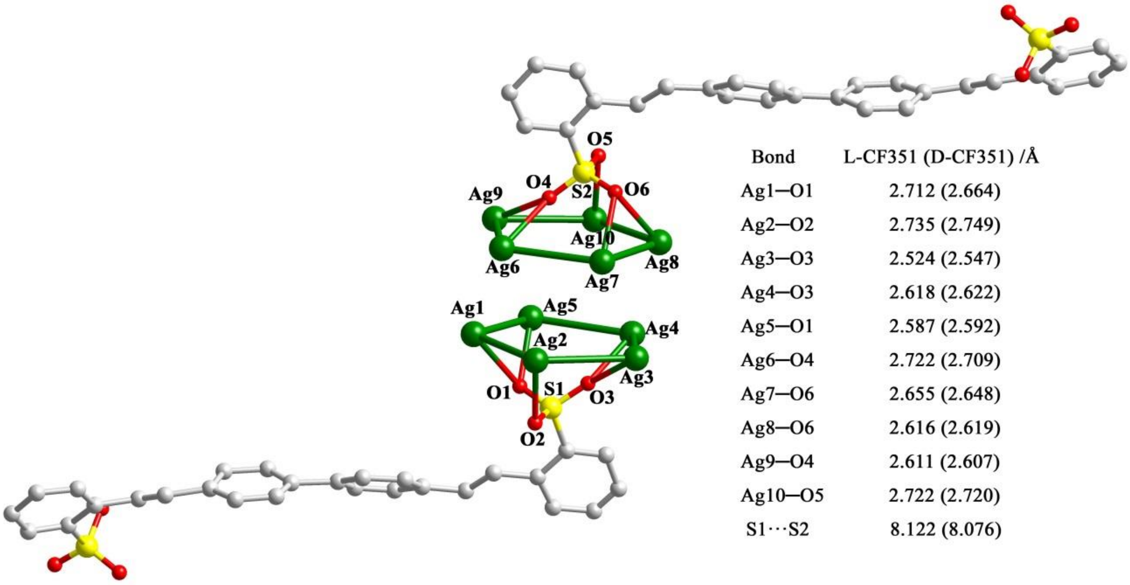


| Bond | L-CF351 (D-CF351) /Å |
|---|---|
| Ag1—O1 | 2.712 (2.664) |
| Ag2—O2 | 2.735 (2.749) |
| Ag3—O3 | 2.524 (2.547) |
| Ag4—O3 | 2.618 (2.622) |
| Ag5—O1 | 2.587 (2.592) |
| Ag6—O4 | 2.722 (2.709) |
| Ag7—O6 | 2.655 (2.648) |
| Ag8—O6 | 2.616 (2.619) |
| Ag9—O4 | 2.611 (2.607) |
| Ag10—O5 | 2.722 (2.720) |
| S1···S2 | 8.122 (8.076) |

**Figure S11.** Bonding mode between $CF351^{2-}$ ligands and $Ag_5$ circles in **L/D-CF351**.

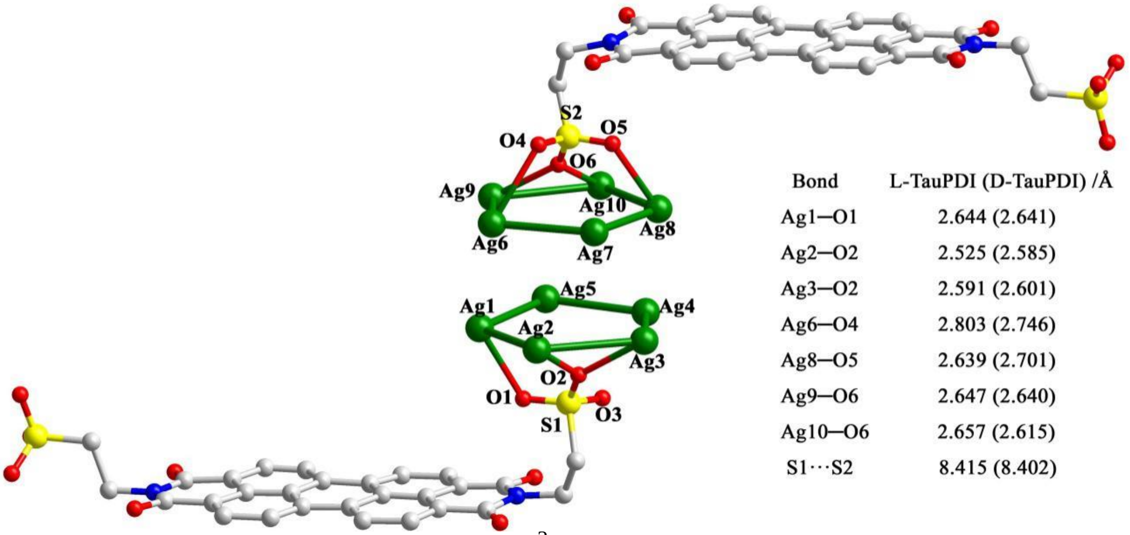


| Bond | L-TauPDI (D-TauPDI) /Å |
|---|---|
| Ag1—O1 | 2.644 (2.641) |
| Ag2—O2 | 2.525 (2.585) |
| Ag3—O2 | 2.591 (2.601) |
| Ag6—O4 | 2.803 (2.746) |
| Ag8—O5 | 2.639 (2.701) |
| Ag9—O6 | 2.647 (2.640) |
| Ag10—O6 | 2.657 (2.615) |
| S1···S2 | 8.415 (8.402) |

**Figure S12.** Bonding mode between $TauPDI^{2-}$ ligands and $Ag_5$ circles in **L/D-TauPDI**.

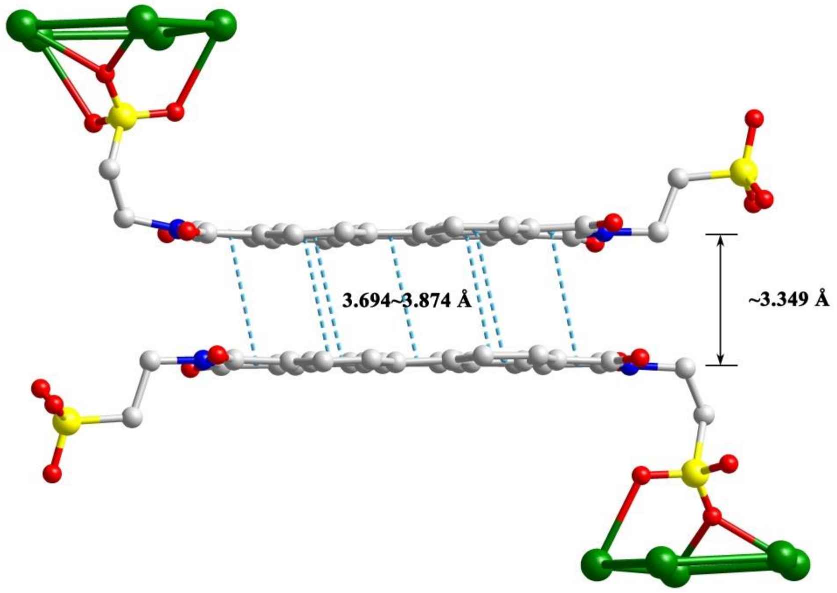


**Figure S13.** π-π stacking interactions between $TauPDI^{2-}$ ligands in **L/D-TauPDI**.

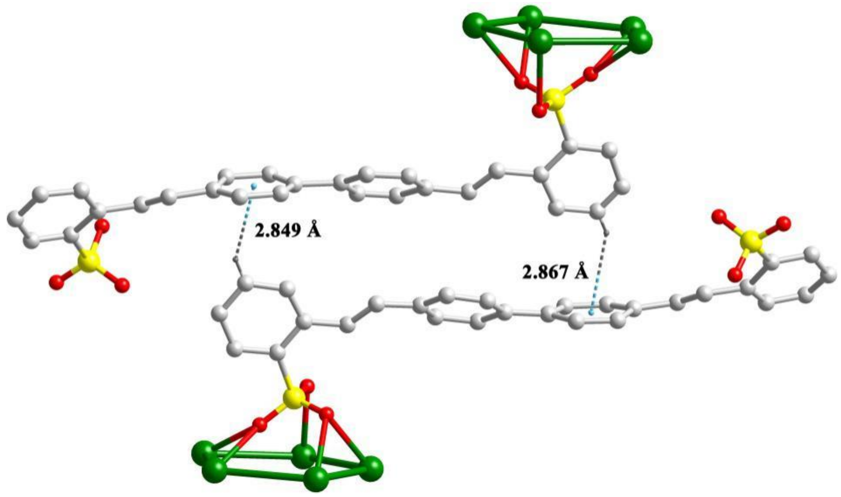


**Figure S14.** C–H···π interactions between $CF351^{2-}$ ligands in **L/D-CF351**.

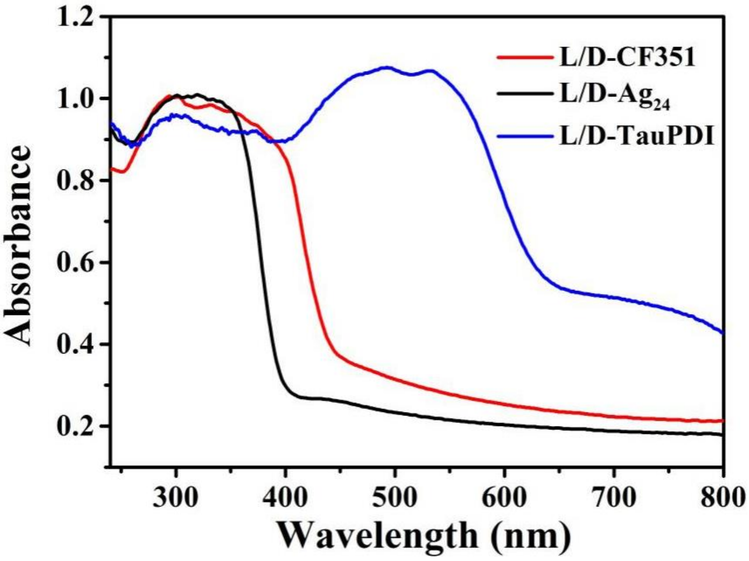


**Figure S15.** Normalized absorption spectra of **L/D-Ag$_{24}$** (black curve), **L/D-CF351** (red curve) and **L/D-TauPDI** (blue curve).

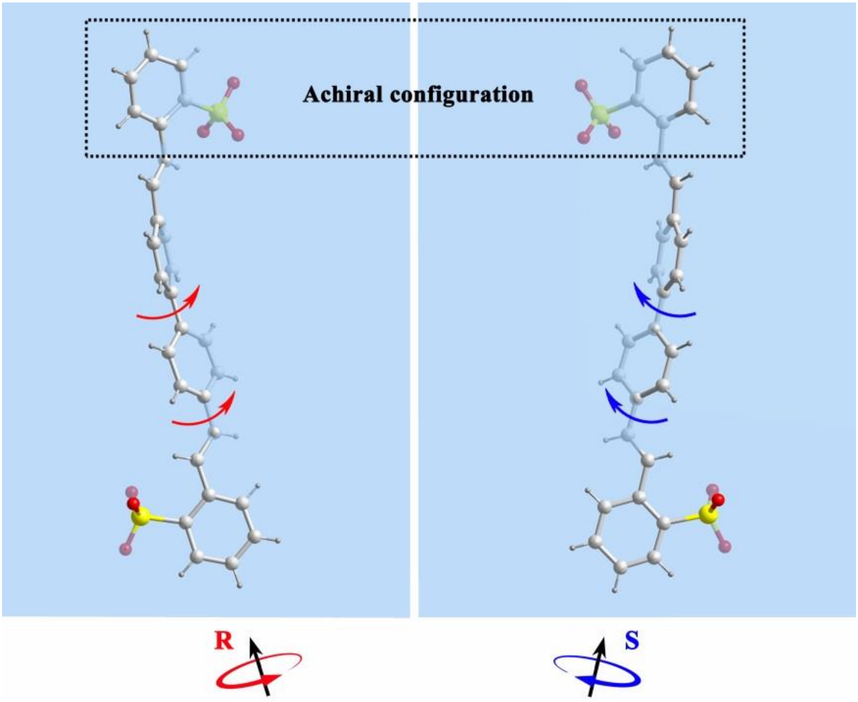

**Figure S16.** The $CF351^{2-}$ molecular configuration in **L/D-CF351** in which the three benzene rings close to the chiral $Ag_{24}$ cluster show R and S configurations, respectively. The phenyl sulfonic groups far away from the chiral $Ag_{24}$ cluster were not induced to chiral configurations.

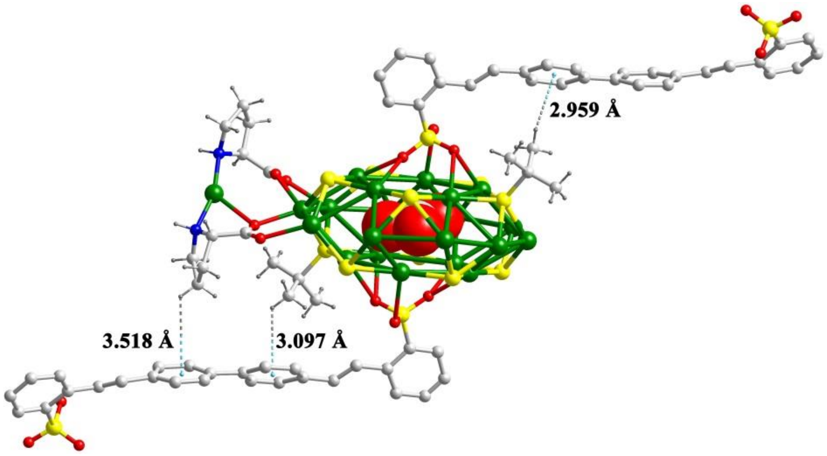


**Figure S17.** C–H···π interactions between *tert*-butyl groups or proline molecules and $CF351^{2-}$ ligands in **L/D-CF351**.

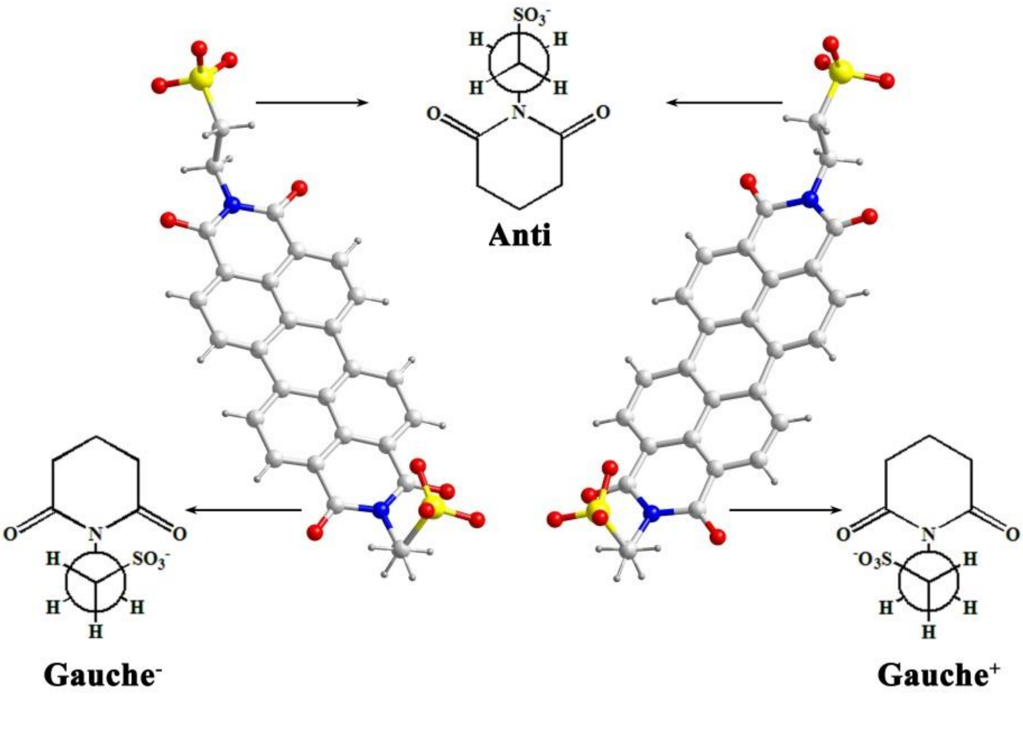

**Figure S18.** The $TauPDI^{2-}$ molecular configuration in **L/D-TauPDI** in which the ethyl sulfonic groups close to the chiral $Ag_{24}$ cluster show $Gauche^{-}$ and $Gauche^{+}$ configurations, respectively. The ethyl sulfonic groups far away from the chiral $Ag_{24}$ cluster show anti configurations.

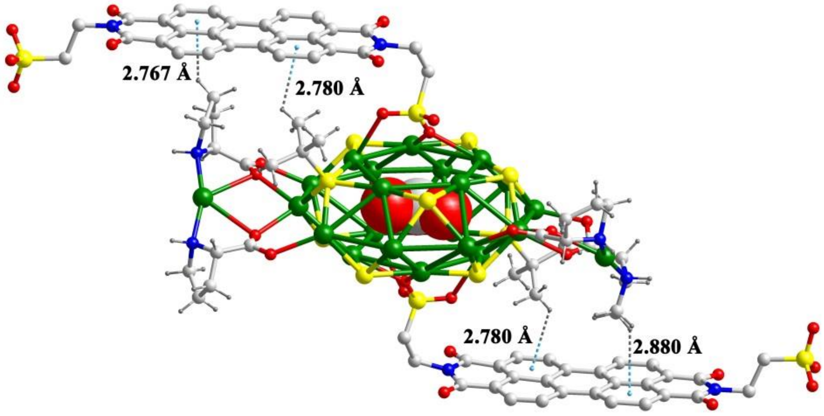


**Figure S19.** C–H···π interactions between *tert*-butyl groups or proline molecules and $TauPDI^{2-}$ ligands in **L/D-TauPDI**.

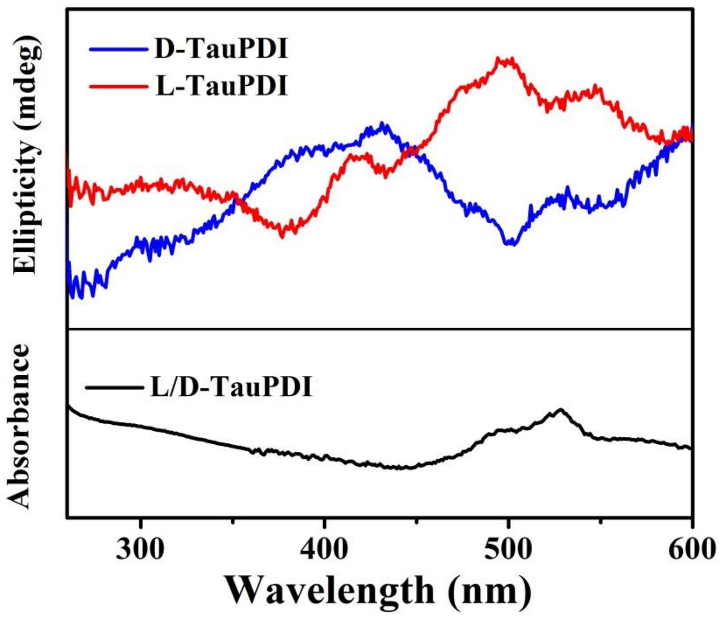

**Figure S20.** CD and UV-vis absorption spectra of water/methanol dispersion solution of **L/D-TauPDI** at room temperature.

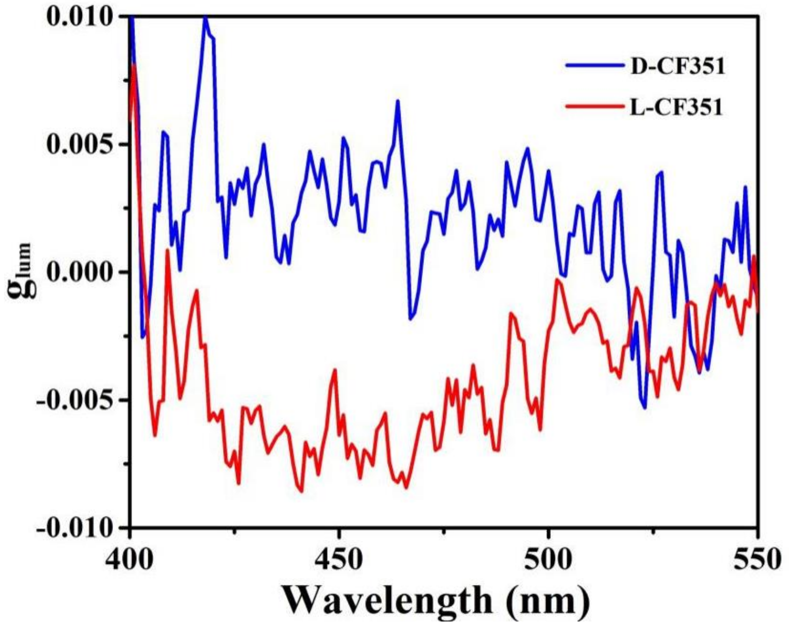


**Figure S21.** Corresponding $g_{lum}$ values of **D-CF351** (blue curve) and **L-CF351** (red curve) in the solid state.

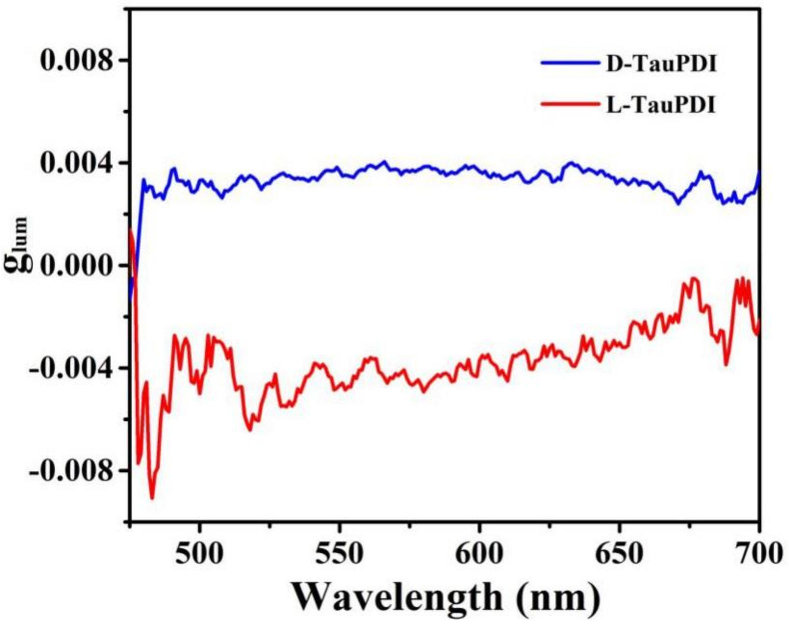


**Figure S22.** Corresponding $g_{lum}$ values of $H_2O/CH_3OH$ dispersion solution of **D-TauPDI** (blue curve) and **L-TauPDI** (red curve).

**Table S1.** Crystal data and structure refinements for **L-CF351** and **D-CF351**.

| | **L-CF351** | **D-CF351** |
|---|---|---|
| **Empirical formula** | $C_{137}H_{196}Ag_{24}N_8O_{32}S_{14}$ | $C_{137}H_{196}Ag_{24}N_8O_{32}S_{14}$ |
| **Formula weight** | 5504.73 | 5504.73 |
| **Temperature/K** | 200(10) | 200(10) |
| **Crystal system** | triclinic | triclinic |
| **Space group** | *P*1 | *P*1 |
| ***a*/Å** | 16.42629(16) | 16.3379(2) |
| ***b*/Å** | 16.44080(17) | 16.4190(2) |
| ***c*/Å** | 20.3039(2) | 20.2852(2) |
| ***α*/°** | 105.7578(10) | 105.7780(10) |
| ***β*/°** | 111.5835(10) | 111.6670(10) |
| ***γ*/°** | 96.2963(8) | 96.2460(10) |
| **Volume/Å$^3$** | 4770.74(10) | 4731.53(10) |
| **Z** | 1 | 1 |
| **ρcalcg/cm$^3$** | 1.916 | 1.932 |
| ***μ*/mm$^{-1}$** | 21.169 | 21.345 |
| **F(000)** | 2682.0 | 2682.0 |
| **Crystal size/mm$^3$** | 0.15× 0.08 × 0.02 | 0.1 × 0.08 × 0.02 |
| **Radiation** | CuK*α* ($\lambda$ = 1.54184 Å) | CuK*α* ($\lambda$ = 1.54184 Å) |
| **2θrange for data collection/°** | 5.746 to 146.712 | 5.754 to 124.974 |
| **Index ranges** | $-14 \leq h \leq 20$, $-20 \leq k \leq 20$, $-25 \leq l \leq 24$ | $-15 \leq h \leq 18$, $-18 \leq k \leq 17$, $-22 \leq l \leq 23$ |
| **Reflections collected** | 52333 | 47666 |
| **Independent reflections** | 23625 [$R_{int}$ = 0.0808, $R_{sigma}$ = 0.0731] | 18870 [$R_{int}$ = 0.0380, $R_{sigma}$ = 0.0469] |
| **Data/restraints/parameters** | 23625/270/1994 | 18870/128/1992 |
| **Goodness-of-fit on F$^2$** | 1.016 | 1.000 |
| **Final R indexes [I>=2*σ* (I)]** | $R_1$ = 0.0720, $wR_2$ = 0.1975 | $R_1$ = 0.0359, $wR_2$ = 0.0942 |
| **Final R indexes [all data]** | $R_1$ = 0.0762, $wR_2$ = 0.2023 | $R_1$ = 0.0379, $wR_2$ = 0.0953 |

| | | |
|---|---|---|
| **Largest diff. peak/hole / e Å$^{-3}$** | 2.39/-1.90 | 1.65/-0.82 |
| **Flack parameter** | 0.063(12) | 0.019(7) |
| **CCDC number** | 1966243 | 1966246 |

$R_1 = \sum||F_o|-|F_c||/\sum|F_o|$. $wR_2 = [\sum w(F_o^2 - F_c^2)^2/\sum w(F_o^2)^2]^{1/2}$

**Table S2.** Crystal data and structure refinements for **L-TauPDI** and **D-TauPDI**.

| | **L-TauPDI** | **D-TauPDI** |
|---|---|---|
| **Empirical formula** | $C_{137}H_{188}Ag_{24}N_{12}O_{40}S_{14}$ | $C_{137}H_{188}Ag_{24}N_{12}O_{40}S_{14}$ |
| **Formula weight** | 5680.70 | 5680.70 |
| **Temperature/K** | 200(10) | 200(10) |
| **Crystal system** | triclinic | triclinic |
| **Space group** | *P*1 | *P*1 |
| ***a*/Å** | 16.3940(3) | 16.4291(4) |
| ***b*/Å** | 17.3042(3) | 17.2780(4) |
| ***c*/Å** | 20.9506(5) | 20.9993(6) |
| ***α*/°** | 75.6808(18) | 75.723(2) |
| ***β*/°** | 77.8940(19) | 77.656(2) |
| ***γ*/°** | 62.8447(17) | 62.680(2) |
| **Volume/Å$^3$** | 5091.54(19) | 5097.2(2) |
| **Z** | 1 | 1 |
| **ρcalcg/cm$^3$** | 1.853 | 1.851 |
| **$\mu$/mm$^{-1}$** | 19.897 | 19.875 |
| **F(000)** | 2766.0 | 2766.0 |
| **Crystal size/mm$^3$** | 0.08 × 0.05 × 0.05 | 0.1 × 0.1 × 0.08 |
| **Radiation** | CuK$\alpha$ ($\lambda$ = 1.54184 Å) | CuK$\alpha$ ($\lambda$ = 1.54184 Å) |
| **2θrange for data collection/°** | 4.38 to 141.128 | 5.844 to 137.68 |
| **Index ranges** | -19 ≤ h ≤ 19, -21 ≤ k ≤ 21, -25 ≤ l ≤ 25 | -19 ≤ h ≤ 19, -20 ≤ k ≤ 20, -25 ≤ l ≤ 25 |
| **Reflections collected** | 120246 | 99863 |
| **Independent reflections** | 35906 [$R_{int}$ = 0.0862, $R_{sigma}$ = 0.0892] | 33183 [$R_{int}$ = 0.0764, $R_{sigma}$ = 0.0786] |
| **Data/restraints/parameters** | 35906/755/2116 | 33183/648/2105 |
| **Goodness-of-fit on F$^2$** | 0.987 | 0.981 |
| **Final R indexes [I>=2$\sigma$ (I)]** | $R_1$ = 0.0854, $wR_2$ = 0.2192 | $R_1$ = 0.0717, $wR_2$ = 0.1857 |
| **Final R indexes [all data]** | $R_1$ = 0.1101, $wR_2$ = 0.2433 | $R_1$ = 0.0899, $wR_2$ = 0.2012 |

| | | |
|---|---|---|
| **Largest diff. peak/hole / e Å$^{-3}$** | 4.38/-1.43 | 3.18/-1.22 |
| **Flack parameter** | 0.062(10) | 0.059(10) |
| **CCDC number** | 1966244 | 1966245 |

$R_1 = \sum||F_o|-|F_c||/\sum|F_o|$. $wR_2 = [\sum w(F_o^2 - F_c^2)^2/\sum w(F_o^2)^2]^{1/2}$

**Table S3.** The main bond lengths of L-$Ag_{24}$.

| Atom-Atom | bond length (Å) | Atom-Atom | bond lengths (Å) |
|---|---|---|---|
| Ag6–S3 | 2.617(5) | Ag5–Ag6 | 3.070(2) |
| Ag6–S10 | 2.647(5) | Ag5–Ag7 | 3.034(2) |
| Ag2–Ag13 | 2.988(2) | Ag5–S10 | 2.426(5) |
| Ag2–Ag12 | 3.323(2) | Ag5–S6 | 2.415(5) |
| Ag2–Ag1 | 3.183(2) | Ag6–Ag18 | 3.315(2) |
| Ag2–S8 | 2.453(5) | Ag6–Ag15 | 3.118(2) |
| Ag2–S9 | 2.457(5) | Ag6–Ag7 | 3.025(2) |
| Ag14–S9 | 2.526(5) | Ag17–Ag12 | 3.081(2) |
| Ag13–Ag12 | 3.129(2) | Ag17–Ag11 | 3.028(2) |
| Ag13–Ag16 | 3.261(3) | Ag17–S5 | 2.413(5) |
| Ag13–S1 | 2.506(5) | Ag17–S1 | 2.411(5) |
| Ag13–S9 | 2.541(5) | Ag17–Ag1B | 3.259(12) |
| Ag4–Ag9 | 3.163(2) | Ag18–Ag15 | 2.970(2) |
| Ag4–Ag8 | 3.148(2) | Ag18–Ag16 | 3.165(3) |
| Ag4–S6 | 2.416(5) | Ag18–S3 | 2.425(5) |
| Ag4–S7 | 2.453(5) | Ag18–S2 | 2.443(5) |
| Ag12–Ag11 | 3.049(2) | Ag15–Ag1 | 3.296(2) |
| Ag12–S8 | 2.584(4) | Ag15–S10 | 2.505(5) |
| Ag12–S1 | 2.648(5) | Ag15–S2 | 2.557(5) |
| Ag19–Ag7 | 3.052(2) | Ag3–Ag4 | 3.247(2) |
| Ag19–Ag8 | 2.961(2) | Ag3–Ag10 | 2.999(2) |
| Ag19–Ag20 | 3.187(6) | Ag3–Ag11 | 3.031(2) |
| Ag19–S3 | 2.433(5) | Ag3–S8 | 2.467(5) |
| Ag19–S4 | 2.515(5) | Ag3–S7 | 2.516(5) |
| Ag1–S10 | 2.435(5) | Ag14–Ag1 | 3.212(2) |
| Ag1–S9 | 2.438(5) | Ag14–Ag16 | 3.249(3) |
| Ag10–Ag11 | 3.006(2) | Ag14–S2 | 2.527(5) |
| Ag9–S4 | 2.462(5) | Ag10–Ag9 | 3.280(2) |
| Ag8–S6 | 2.496(5) | Ag10–Ag20 | 3.113(11) |
| Ag8–S4 | 2.480(5) | Ag10–S5 | 2.484(5) |
| Ag8–O24 | 2.253(12) | Ag10–S7 | 2.464(4) |
| Ag20–S5 | 2.381(6) | Ag10–O23 | 2.255(13) |
| Ag20–S4 | 2.393(7) | Ag7–Ag8 | 3.060(2) |
| Ag16–S1 | 2.426(5) | Ag7–S3 | 2.543(5) |
| Ag16–S2 | 2.413(5) | Ag7–S6 | 2.638(5) |
| Ag28–N12 | 2.180(14) | Ag11–S8 | 2.542(5) |

| | | | |
|---|---|---|---|
| Ag2A–N6 | 2.190(14) | Ag11–S5 | 2.656(5) |
| Ag2A–N5 | 2.191(15) | Ag11–O21 | 2.261(12) |
| Ag9–Ag20 | 3.108(12) | Ag9–Ag8 | 3.258(2) |
| Ag9–S7 | 2.470(5) | | |

**Table S4.** The main bond lengths of **L-CF351**.

| Atom-Atom | bond length (Å) | Atom-Atom | bond lengths (Å) |
|---|---|---|---|
| Ag1–Ag3 | 3.012(2) | Ag4–O20 | 2.369(12) |
| Ag1–Ag9 | 3.297(2) | Ag5–Ag12 | 3.273(2) |
| Ag1–Ag18 | 3.054(2) | Ag5–S5 | 2.464(5) |
| Ag1–Ag20 | 2.988(2) | Ag5–S7 | 2.478(4) |
| Ag1–S3 | 2.468(4) | Ag5–O29 | 2.53(2) |
| Ag1–S11 | 2.494(4) | Ag5–O34 | 2.44(3) |
| Ag1–O10 | 2.251(11) | Ag6–Ag12 | 3.159(2) |
| Ag2–Ag6 | 3.192(2) | Ag6–Ag13 | 2.906(2) |
| Ag2–Ag8 | 3.271(2) | Ag6–Ag16 | 3.228(2) |
| Ag2–Ag12 | 3.108(2) | Ag6–S1 | 2.485(4) |
| Ag2–S7 | 2.525(4) | Ag6–S9 | 2.547(4) |
| Ag2–S9 | 2.535(4) | Ag7–Ag11 | 3.304(2) |
| Ag2–O15 | 2.368(12) | Ag7–Ag17 | 3.151(2) |
| Ag3–Ag11 | 3.298(2) | Ag7–Ag19 | 3.136(2) |
| Ag3–Ag20 | 3.169(2) | Ag7–S2 | 2.504(5) |
| Ag3–S3 | 2.535(5) | Ag7–S8 | 2.440(4) |
| Ag3–S12 | 2.481(5) | Ag8–Ag13 | 3.380(2) |
| Ag4–Ag5 | 2.958(2) | Ag8–S8 | 2.443(5) |
| Ag4–Ag8 | 3.059(2) | Ag8–S9 | 2.460(5) |
| Ag4–Ag19 | 3.304(2) | Ag9–Ag10 | 3.178(2) |
| Ag4–S7 | 2.537(4) | Ag9–Ag11 | 3.065(2) |
| Ag4–S8 | 2.514(5) | Ag9–Ag18 | 3.127(2) |
| Ag14–S5 | 2.451(4) | Ag9–S3 | 2.459(4) |
| Ag14–S6 | 2.496(4) | Ag9–S6 | 2.460(4) |
| Ag14–O7 | 2.524(13) | Ag10–Ag11 | 3.215(2) |
| Ag15–Ag16 | 3.222(2) | Ag10–Ag14 | 3.014(2) |
| Ag15–Ag20 | 3.068(2) | Ag10–Ag17 | 3.006(2) |
| Ag15–S1 | 2.467(4) | Ag10–S2 | 2.477(5) |
| Ag15–S11 | 2.469(4) | Ag10–S6 | 2.496(5) |
| Ag16–Ag20 | 3.107(2) | Ag11–S2 | 2.466(4) |
| Ag16–S1 | 2.534(4) | Ag11–S3 | 2.471(4) |
| Ag16–S12 | 2.580(5) | Ag12–Ag15 | 3.274(2) |
| Ag17–Ag19 | 3.051(2) | Ag12–S1 | 2.486(4) |
| Ag17–S2 | 2.512(4) | Ag12–S7 | 2.466(4) |
| Ag17–S5 | 2.524(5) | Ag13–Ag16 | 3.332(2) |

| | | | |
|---|---|---|---|
| Ag18–S6 | 2.465(5) | Ag13–S9 | 2.445(4) |
| Ag18–S11 | 2.439(5) | Ag13–S12 | 2.424(4) |
| Ag19–S5 | 2.590(4) | Ag14–Ag17 | 2.993(2) |
| Ag19–S8 | 2.605(5) | Ag14–Ag18 | 3.271(2) |
| Ag19–O24 | 2.298(13) | Ag21–N5 | 2.161(16) |
| Ag20–S11 | 2.551(4) | Ag22–N4 | 2.171(19) |
| Ag20–S12 | 2.510(4) | Ag23–O19 | 2.511(17) |
| Ag20–O13 | 2.480(13) | Ag24–O21 | 2.600(17) |
| Ag20–O19 | 2.317(15) | Ag24–N2 | 2.212(16) |
| Ag21–N1 | 2.140(18) | | |

**Table S5.** The main bond lengths of **L-TauPDI**.

| **Atom-Atom** | **bond length (Å)** | **Atom-Atom** | **bond lengths (Å)** |
|---|---|---|---|
| Ag1–S6 | 2.448(7) | Ag6–Ag17 | 3.012(3) |
| Ag1–S7 | 2.510(7) | Ag6–Ag15 | 3.351(3) |
| Ag1–Ag16 | 2.976(3) | Ag6–Ag13 | 3.359(3) |
| Ag1–Ag4 | 3.020(3) | Ag7–S2 | 2.498(7) |
| Ag1–Ag15 | 3.085(3) | Ag7–S4 | 2.546(6) |
| Ag1–Ag14 | 3.329(3) | Ag7–Ag8 | 2.972(3) |
| Ag2–S9 | 2.491(6) | Ag7–Ag19 | 3.067(3) |
| Ag2–S3 | 2.509(6) | Ag7–Ag12 | 3.147(3) |
| Ag2–Ag13 | 3.033(3) | Ag8–S5 | 2.392(6) |
| Ag2–Ag10 | 3.072(3) | Ag8–S4 | 2.463(6) |
| Ag2–Ag18 | 3.130(3) | Ag8–Ag12 | 3.302(3) |
| Ag3–S10 | 2.486(7) | Ag9–S8 | 2.420(7) |
| Ag3–S8 | 2.499(7) | Ag9–S6 | 2.460(7) |
| Ag3–Ag17 | 2.998(3) | Ag9–Ag14 | 3.115(3) |
| Ag3–Ag6 | 3.043(3) | Ag9–Ag11 | 3.344(3) |
| Ag3–Ag14 | 3.145(3) | Ag10–S9 | 2.431(6) |
| Ag3–Ag9 | 3.146(3) | Ag10–S4 | 2.441(6) |
| Ag4–S5 | 2.497(7) | Ag10–Ag11 | 3.337(3) |
| Ag4–S6 | 2.542(7) | Ag11–S9 | 2.403(6) |
| Ag4–Ag16 | 3.213(3) | Ag11–S8 | 2.451(7) |
| Ag4–Ag9 | 3.236(3) | Ag11–Ag17 | 3.101(3) |
| Ag4–Ag8 | 3.372(3) | Ag11–Ag18 | 3.286(3) |
| Ag5–S3 | 2.514(6) | Ag12–S5 | 2.554(7) |
| Ag5–S4 | 2.519(6) | Ag12–S2 | 2.573(6) |
| Ag5–Ag19 | 3.145(3) | Ag12–Ag16 | 3.045(3) |
| Ag5–Ag10 | 3.250(3) | Ag12–Ag20 | 3.294(5) |
| Ag5–Ag7 | 3.254(3) | Ag13–S1 | 2.461(6) |
| Ag6–S1 | 2.431(7) | Ag13–S3 | 2.480(6) |
| Ag6–S10 | 2.501(7) | Ag13–Ag19 | 3.307(3) |

| | | | |
|---|---|---|---|
| Ag17–S1 | 2.503(6) | Ag14–S6 | 2.446(7) |
| Ag17–S8 | 2.557(7) | Ag14–S10 | 2.464(7) |
| Ag17–Ag18 | 3.062(3) | Ag14–Ag15 | 3.119(3) |
| Ag18–S9 | 2.555(6) | Ag15–S7 | 2.410(7) |
| Ag18–S1 | 2.578(7) | Ag15–S10 | 2.447(7) |
| Ag19–S2 | 2.440(6) | Ag15–O6 | 2.591(16) |
| Ag19–S3 | 2.440(6) | Ag15–Ag20 | 3.348(4) |
| Ag19–Ag20 | 3.317(4) | Ag16–S5 | 2.521(6) |
| Ag20–S7 | 2.383(8) | Ag16–S7 | 2.548(7) |
| Ag20–S2 | 2.411(7) | Ag16–Ag20 | 3.168(5) |

## References

[S1] CrysAlis$^{Pro}$ Version 1.171.36.31. (2012). Agilent Technologies Inc. Santa Clara, CA, USA.

[S2] L. Krause, R. Herbst-Irmer, G. M. Sheldrick, D. Stalke, *J. Appl. Cryst.* **2015**, *48*, 3.

[S3] G. M. Sheldrick, *Acta Cryst. A* **2008**, *64*, 112.

[S4] O. V. Dolomanov, L. J.Bourhis,R. J.Gildea, J. A.-K. Howard, H. *J.* Puschmann, *Appl. Cryst.* **2009**, *42*, 339.

[S5] G. M. Sheldrick, *Acta Cryst. C* **2015**, *71*, 3.

[S6] A. L. Spek, *Acta Cryst. C* **2015**, *71*, 9.
